# A Modern Operative Equipment

**Published:** 1904-12-15

**Authors:** E. A. Honey


### A MODERN OPERATIVE EQUIPMENT.

BY E. A. HONEY, D.D.S.

Read before the Michigan Dental Association, June 29, 1904.

When this subject was assigned to me by your worthy trustee having this part of the program in charge, it appeared to be quite an attractive task, for I thought a couple of hours consultation with a modern catalogue of dental appliances would immediately furnish me with abundant material for the subject in hand. After a little careful thought, however, I soon found if that plan were followed I would be in much the same position as the ruralite who, for the first time, ordered his meal from a printed bill of fare; having measured a goodly third or half of the entire bill, instructed the waiter to bring on that amount, only to observe some time later that the gentleman at the adjoining table was enjoying a real meal while he was filled to the brim with consomme.

A moment's reflection will be sufficient to convince any one present that a complete description of what we have been pleased to call "A Modern Operative Equipment," can only be general in character if it is to have general application, inasmuch as a list of appliances, sufficient in length to meet the requirements of all would keep you here until to-morrow morning in the reading alone. This would bring together such a jumbled mass of names that there would be more difficulty in selecting such as would meet your individual wants, than the finding of your own hat at the close of a Lincoln Club banquet.

The selection of an operating outfit, complete and modern, requires careful consideration, and the more generous your bank account, the greater should be the discrimination in making your purchases. Much care should be exercised in eliminating such appliances as have no real value. Nothing should be placed in the operating room that does not contribute to the convenience of the operator or the comfort of the patient, for it is an easy matter to fill your cabinets and work benches with appliances, modern so far as recent invention goes, and yet contribute little or nothing to the quality of your work or the facility of your operation.

Another difficulty just at this point is in knowing just where to draw the line between the appliances that properly belong to the operating room and those of the laboratory; but inasmuch as many offices in the larger towns and cities fail in space for laboratories, we will suppose for the time being that the laboratory work, such as vulcanizing, etc., is done out of the office entirely and will proceed to furnish the operating room with this in view.

Taking for granted that we are provided with a room not less than ten or twelve feet in width by about fourteen or more in length, with a window of good dimensions especially in height and facing the north or east, we will begin in an imaginary way to place a few of the appliances.

The floor should be of either tile or hard wood with as high a polish as it is possible to maintain, as no floor should have any covering that can not be removed daily and cleaned. The walls and ceilings should be made attractive by paper or water color of such tints as are restful to the eye, together with a few well-chosen pictures, but should be kept free from anatomical charts, diplomas and other such form of decorations, all of which are essential enough however in their place, but should not be given room on the walls of your operating room.

It is needless to say that the chair, from a sanitary standpoint if nothing else, should have a disc base and should be the one you have found to yield the greatest convenience to the operator and comfort to the patient. It should be free from all upholstering, finished in cane, as this will be found to meet the requirements from a standpoint of cleanliness and will meet with the unqualified approval of the vast majority of your patrons.

The fountain spittoon should be furnished with a glass or porcelain bowl with the metal parts of polished brass. The dental profession are to be greatly congratulated for the beautiful and convenient appliances of this character with which it is supplied.

The next thing to be considered is the dental engine, and the preference among our best operators seems to be almost unanimous in favor of the all-cord. The only regret to me is, that it presents such a display of belts, pulleys and nickel work and it will be a boon to the profession when some one will invent an engine furnishing power direct to the hand piece and eliminating to the greatest possible extent these objections.

The dental cabinet should be chosen from the many good ones now on the market, that presents the greatest convenience possible and is entirely free from carving and other attempt at cheap ornamentation, which simply serve as dust catchers and soon become tiresome to the eye. This should apply with equal force to all the furniture in the room, which should be of the same wood and finish. A roll-top work bench should be provided for crown and bridge-work and piped for gas and compressed air and wired conveniently for connection with the polishing lathe.

No office is complete without an electric switchboard, to which may be connected the engine, furnace, lathe, annealer, hot-air syringe, cautery, mouth lamp and such other electrical appliances as may be needed to meet the individual wants. This switchboard would be better covered or enclosed in a cabinet admitting the light from the side foward the window, and provided with a door on the opposite side through which we may conveniently operate the switches and yet conceal the entire appliance from the constant view of the patient. In fact, it should be the constant study of the operator to have as little machinery in evidence as possible.

If the building is not supplied with a compressed-air system, it must be provided in some other manner which may be either the hydraulic or electric pump. Of the latter form there are one or two on the market that meet the requirements fairly well. The pipes leading from the receiver and distributing the air to the most convenient points for use should be concealed under the floors or in the partitions where such is possible. Attached to your switchboard or close at hand should be provided a gauge which will allow you to instantly control the air pressure to meet the requirements in the variety of cases for which it is used. I would like to emphasize the importance of this particular feature in the use of compressed air, as a strong blast of either hot or cold air on a sensitive tooth produces a decided shock that should be carefully avoided.

So far as I know, the dental dealers do not furnish a plaster bench suitable for an operating room, yet it is very essential as no one should be obliged to step many feet from his chair for anything in securing impressions for crowns, bridges, etc. A bench for this purpose may be easily designed to meet all requirements and yet be in keeping with the surroundings. In addition to the above might be added a water heater where hot water can be supplied on a moment's notice all day; some form of appliance for administering nitrous oxide, which should be kept concealed from the view of the patients when not in use. I place this latter appliance in the operating room for the reason that its use is by no means confined as an anesthetic for extracting.

The above list of general appliances is by no means complete, yet I think it contains nothing that could possibly detract from the convenience of the operator.

As to the smaller instruments, of which there is an endless variety of makes, I will not attempt to even outline, for it must be apparent to all of you that such appliances are gathered together one at a time as the necessity demands, on much the same principle as one builds up a library, and as much individuality is shown in the one case as in the other. A complete list of such instruments would inevitably contain much that would be as useless as the wooden dummies with which the newly-rich man filled his book shelves.

---

